# A Temporal Comparison of 50 Years of Australian Scuba Diving Fatalities

**DOI:** 10.3390/ijerph22071148

**Published:** 2025-07-19

**Authors:** John M. Lippmann

**Affiliations:** 1 Australasian Diving Safety Foundation, Canterbury, VIC 3800, Australia; johnl@adsf.org.au; 2 Department of Public Health and Preventive Medicine, Monash University, Melbourne, VIC 3168, Australia; 3 The Royal Lifesaving Society—Australia, Sydney, NSW 2700, Australia

**Keywords:** diving, cardiac arrhythmia, deaths, drowning, fatalities, obesity, scuba

## Abstract

Australian scuba fatalities over 50 years were examined to determine temporal changes over two consecutive periods, 1972–1999 and 2000–2021. The Australasian Diving Safety Foundation database and National Coronial Information System were searched to identify scuba deaths from 1972 to 2021. Historical data, police and witness reports, and autopsies were recorded and comparisons made between the two periods. Of 430 total deaths, 236 occurred during 1972–1999 and 194 during 2000–2021, with average annual fatalities of 8.4 and 8.8, respectively. The proportion of males reduced (83% to 76%) and median ages rose (33 to 47 years) with a large rise in the percentage of casualties among people aged 45 years or older (24% to 57%). There were increases in certified divers (64% to 81%) and in the proportion of divers who were with a buddy at the time of their incident (17% to 27%), as well as a decrease in out-of-gas incidents (30% to 25%). A reduction in primary drowning (47% to 36%) was accompanied by more than a doubling of cardiac-related disabling conditions (12% to 26%). The substantial increase in casualties’ ages and of the proportions of casualties aged 45 or more and of females between the periods indicate the inclusion of a broader cohort of participants and ageing of longtime divers. The reduction in primary drowning was likely due to increased training and improvements in equipment, particularly BCDs and pressure gauges. The rise in cardiac-related deaths was due to an older and more obese cohort. Improved health education and surveillance and improved dive planning are essential to reduce such deaths.

## 1. Introduction

With its vast coastline, wide variety of marine life, from tropical in the north to temperate further south, and numerous shipwrecks throughout, Australia offers wonderful opportunities for scuba diving for both locals and tourists. Estimates vary considerably, but a recent government survey suggests that over more recent years, at least 57,000 Australians have gone scuba diving annually [1]. There are also many overseas tourists who dive, mainly on the Great Barrier Reef.

Since scuba diving is conducted in a hostile environment, there are a variety of associated risks. These include risks from the environment itself, the equipment needed to survive underwater, and the participant’s suitability—including their training and experience, physical medical and psychological health, and attitude. It has been estimated that the fatality rate in divers who are Australian residents is around 0.48 deaths per 100,000 dives, although the risk can vary between locations due to general conditions and practices [2].

Recreational scuba diving emerged in Australia around the early 1950s, and, although some early fatality reports became available from 1967, formal and regular reports of Australian scuba fatalities began in 1972 [3] and have continued [4,5,6]. Certain aspects of diving have changed over time, including equipment, training, and the demographics and characteristics of the divers. It is important to periodically review such changes to assess their impact, if any, on diving safety.

The aim of this study is to review scuba fatalities over 50 years and determine temporal changes by comparing two consecutive periods, 1972–1999 and 2000–2021.

## 2. Materials and Methods

This is a retrospective case series of recorded scuba diving deaths in Australia from 1 January 1972 to 31 December 2021, inclusive. Ethics approval for the collection and reporting of these data was received from the Victorian Justice Human Research Ethics Committee to access the National Coronial Information System (NCIS) (approval number CF/21/18434). A search was made of the NCIS [7] for scuba-related deaths from 1 July 2000 (from when cases are listed) to 31 December 2021 and these were matched with data obtained from other sources, including additional coronial documents and police and media reports. Earlier data were obtained from the Australasian Diving Safety Foundation fatality database (ADSF) [8] and Project Stickybeak Reports [9,10].

**Figure 1:**
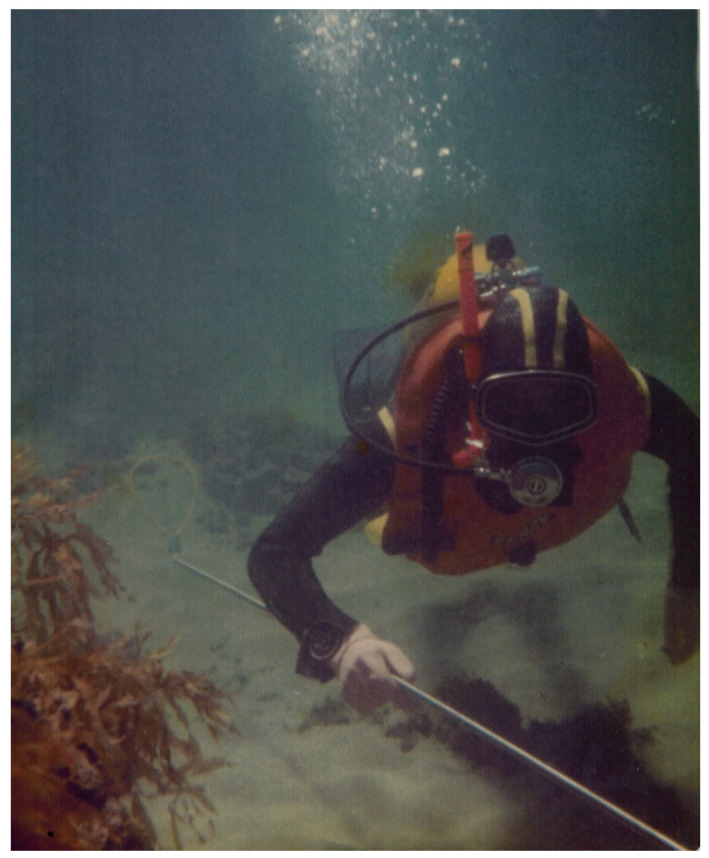
A (young) diver in 1973.

Information on demographics, health, certification, experience, dive circumstances, and forensic investigations were extracted. A chain of events analysis was performed for each case, focusing on predisposing conditions, incident triggers, disabling conditions, and reported causes of death using existing templates [11]. Data for the periods 1972–1999 (Figure 1) and 2000–2021 were compared.

Descriptive analyses based on means and standard deviations or medians and ranges were conducted, as appropriate, using SPSS Version 30.0.0.0 (IBM Armonk, NY, USA; 2024). The level of statistical significance assumed was *p* = 0.05.

## 3. Results

**Figure 2:**
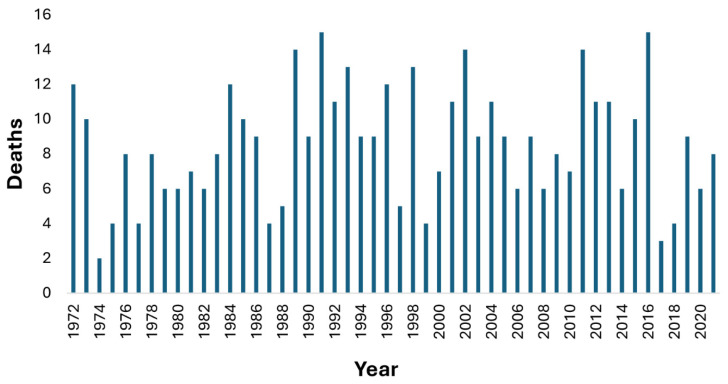
Annual diving fatalities from 1972 to 2021.

There were 430 recorded scuba-related deaths over the 50-year period comprising 343 (80%) males and 87 (20%) females, with 236 deaths from 1972 to 1999, and 194 from 2000 to 2021, inclusive. There was no clear trend, with peaks and troughs in deaths throughout, and with an average of 8.4 deaths/year in 1972–1999 and 8.8 deaths/year in 2000–2021 (Figure 2).

### 3.1. Demographics

**Figure 3:**
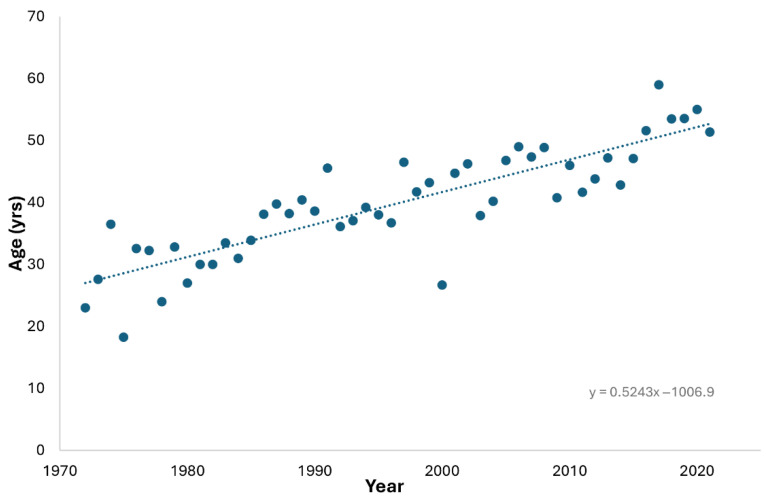
Average age of scuba fatality casualties from 1972 to 2021.

There was a steady increase in the age of casualties over the 50-year period, rising by approximately 5 years each decade (Figure 3).

From 1972 to 1999, the median (IQR) age of the casualties was 33 (25, 44) years (range = 13–70 years) and 196 (83%) of the casualties were males. By comparison, from 2000 to 2021, the median age of fatalities increased to 47 (37, 55) years (range 17–72 years), with 148 (76%) being males. There was no significant difference in the ages between sexes for both periods.

**Figure 4:**
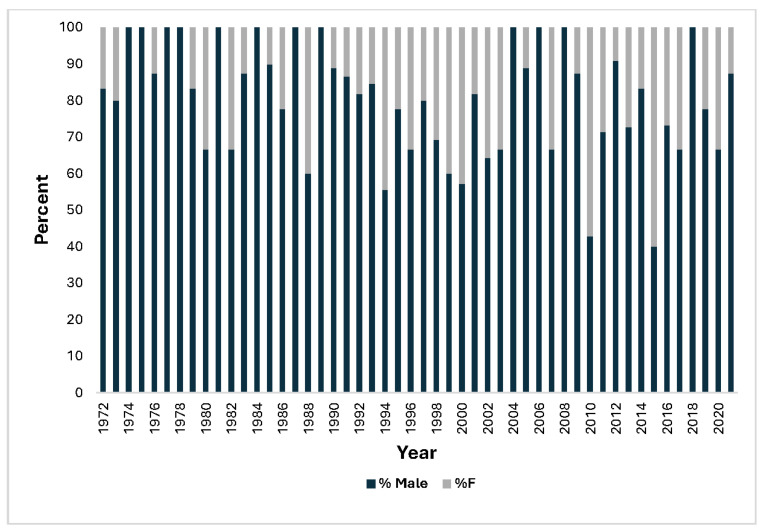
Relative proportions of male and female fatalities annually.

From 1972 to 1999, 24% of casualties were aged 45 years or older, increasing to 57% from 2000 to 2021. The proportion of female casualties increased over time (Figure 4).

### 3.2. Training

In the period from 1972 to 1999, 152 (64%) of the divers were certified, 19 (8%) were undergoing training when the accident occurred, 48 (20%) were uncertified, and there was no relevant information on the remainder. From 2000 to 2021, 158 (81%) casualties were certified, 12 (6%) were undergoing training, and 10 (5%) were uncertified, with 14 (7%) unreported.

### 3.3. Breathing Gas Depletion

From 1972 to 1999, 70 (30%) casualties had exhausted their breathing gas supply, whereas this dropped to 49 (25%) in the later period.

### 3.4. Buddy Circumstances

From 1972 to 1999, 26 (11%) casualties had set out solo and only 41 (17%) divers were with a buddy during their incident. 117 (50%) had separated before the incident and 43 (18%) during, with the remainder unreported. By comparison, from 2000 to 2021, 24 (12%) casualties had set out alone, 53 (27%) were with a buddy at the time of their demise, 77 (40%) had separated before their incident, 29 (15%) had separated during their incident, and the remainder were unreported.

### 3.5. Weight Belt and Buoyancy Compensator Device (BCD) Circumstances

Based on the available data, from 1972 to 1999, 27/201 (13%) had ditched their weights, and 34/150 (23%) had inflated or partly inflated their BCD, only 4 of whom had also ditched their weights. 57 victims had not been wearing a BCD. By comparison, from 2000 to 2021, 26/151 (17%) had ditched their weights and 44/137 (32%) were found with inflated or partly inflated BCDs, 11 of whom had also ditched their weights. Three victims were known not to have been wearing a BCD.

### 3.6. Predisposing Factors (PFs)

Certain factors can predispose to an incident (whether fatal or non-fatal) while diving and these include health-related factors, such as poor fitness or a pre-existing medical condition, inexperience, adverse sea conditions, and the effects of alcohol or drugs, among others. In many cases, there are multiple PFs present which may have contributed to the incident. For example, an inexperienced diver with a faulty pressure gauge who goes diving in unsuitable conditions has multiple vulnerabilities from the outset.

#### 3.6.1. 1972–1999

The main PFs during this period were inexperience, planning, equipment, and health factors, in that order.

Inexperience was identified as a contributor to at least 94 (40%) incidents. Poor planning predisposed to 78 (33%) of the fatalities. The main planning problem was the decision to dive in adverse conditions which was identified in 40 cases. These conditions included rough seas, strong currents, or strong surge, pre-existing or the potential for very poor visibility (e.g., silting in caves or wrecks), and very cold water. The decision to set out solo or planned separations likely contributed to 35 deaths. Other planning shortcomings involved failure to account for hazards (e.g., outlets in dams) and poor pre-planning of likely air usage.

Equipment-related PFs likely contributed to 71 (30%) of the deaths. The most frequent was the lack of a buoyancy control device (BCD) which was identified in 20 cases, mainly early in the period when they were relatively rare. Faulty BCDs were also identified in nine cases. Other problems included overweighting (15), regulator or tank valve faults (10), absent or overtight wetsuits (9), faulty or absent pressure gauges (5), and absent fins (4). Failure to use guidelines when diving in silty caves or wrecks was identified in four accidents leading to ten deaths. Notably, the equipment faults mainly resulted in primary drownings (52) and pulmonary barotrauma/arterial gas embolism (PBT/CAGE, 14).

Health-related PFs were identified in 56 (24%) of the fatalities and likely directly contributed to 43 (18%) of the deaths. The main factors were cardiac-related, which were identified in 30 deaths. Twenty of these involved severe ischaemic heart disease (IHD), often undiagnosed. Body mass index (BMI) was rarely recorded for casualties during this period.

#### 3.6.2. 2000–2021

Health-related predisposing factors were the most common in this period, identified in 102 (53%) of the casualties, and were likely directly contributory to 78 (40%) of the deaths, with at least 50 of these predispositions being cardiac-related, predominantly IHD. Notably, of 163 casualties whose BMIs were recorded, 70 (43%) were classified as obese (BMI ≥ 30 kg·m^−2^) or severely obese (BMI ≥ 35 kg·m^−2^).

Inexperience was identified as a likely contributor to 58 (30%) deaths, and planning inadequacies featured in at least 56 (29%) cases, again mainly involving setting out in adverse conditions (25), and diving alone or planned separations (23).

Pre-existing equipment issues were identified in 40 (21%) of the deaths with several divers having multiple issues. These included faulty (often high-reading) gauges (6), faulty demand valves (6), gross overweighting (6), faulty BCDs (3), leaking cylinder valves (3), poorly/incorrectly configured cylinder and regulator combinations (3), incorrect gas mix/contamination (3), and faulty closed-circuit rebreather sensors, among others. Two divers were not wearing wetsuits in cold water, and one diver became incapacitated as his wetsuit was too tight.

### 3.7. Disabling Conditions

The disabling condition (DC) is defined as an injury or condition directly responsible for death, or for incapacitation followed by death, from drowning [11]. For example, consider a diver with a faulty pressure gauge (predisposing factor) who runs out of air, makes an emergency ascent failing to exhale sufficient air as it expands during ascent, suffers an arterial gas embolism (AGE), become unconscious, and subsequently drowns. In this case, the DC is arterial gas embolism, and the cause of death (COD) is drowning.

When investigating diving fatalities (as well as other aquatic fatalities), it is often more informative to consider the DC rather than the COD as many aquatic incidents ultimately result in drowning. In diving, drownings often occur secondary to cardiac events, AGE, trauma, and a variety of other conditions.

**Figure 5:**
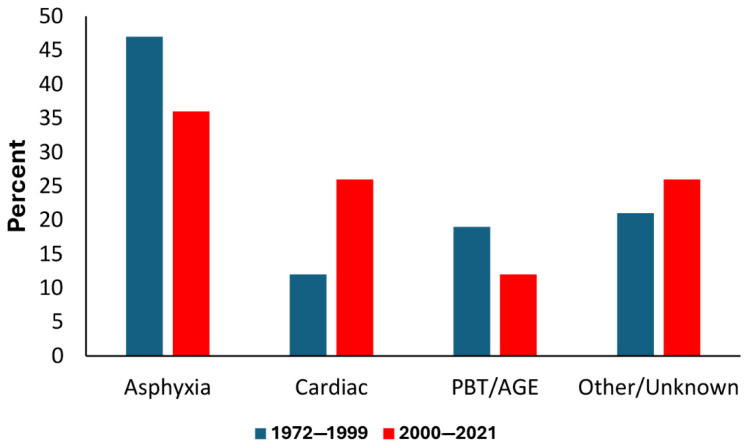
Comparison of disabling conditions for the periods 1972–1999 and 2000–2021. Asphyxia = primary drowning; PBT/AGE = pulmonary barotrauma/arterial gas embolism.

A comparison of the DCs for the two periods is shown in Figure 5. Note the reduction in asphyxia (i.e., primary drowning) and increase in cardiac-related DCs. There was also a reduction in cases of lung over-expansion injuries (pulmonary barotrauma) and associated arterial gas embolisms (PBT/AGEs).

Overall, there were many changes in diver and diving characteristics between periods which are summarised in Table 1.

## 4. Discussion

There was no obvious trend in the annual fatalities’ frequencies, with peaks and troughs throughout. However, the ages of scuba fatality casualties increased substantially between the periods, and there was an increase in the proportion of females. Fewer casualties were uncertified, and out-of-gas incidents reduced. Although the proportion of solo divers was similar, fewer divers who set out with buddies were alone at the time of their incident. Health-related predisposing factors increased over time, while deaths associated with inexperience and equipment-related issues declined. There was a rise in cardiac-related deaths, while those arising from primary drowning and arterial gas embolism reduced.

### 4.1. Annual Fatalities

Despite the strong growth of scuba diving in some parts of the world, there has been a general reduction in entry-level diver certifications in Australia since the late 1980s/early 1990s which was a busy period for diving in Australia [12]. There is also no reliable indication of growth in the number of active divers over much of the more recent period [2]. Therefore, it is unsurprising that, based on activity level alone, the mean annual fatalities have not grown significantly.

### 4.2. Demographics

The increase in ages of the casualties is consistent with longtime divers ageing and a broader and often older population cohort being attracted to the activity. Government sporting surveys for 2001–2010 estimated that 30% of Australian divers were aged over 45 years [2]. With age comes an increasing prevalence of chronic disease and its implications for diving safety, as discussed later [13].

The rise in the proportion of female casualties is consistent with their increased participation over time. PADI data for Australia for 1972–1999 and 2000–2021 indicate that 33% and 39%, respectively, of certifications were for females [14], so, given that 17–24% of deaths were in females during those periods, females are underrepresented in dive fatalities.

### 4.3. Training

Scuba certification agencies became active in Australia from around the early 1970s, and more emerging divers began to seek and receive formal training. The increased availability of training accounts for the substantial reduction in fatality casualties who were uncertified. However, there remains too many untrained casualties, so the importance of appropriate training still needs to be reinforced.

Practical training requirements in Australia have changed considerably over time, with a progressive reduction in open water training time and an increase in student-to-instructor ratios. In addition, a fitness-to-dive assessment prior to certification was common until about 2013, but has been subsequently replaced by a questionnaire, as used in most other countries and consistent with the ISO scuba training standards [15]. Currently, any effect of such changes on diving safety is unclear, especially in the context of increased cardiac disabling conditions in divers with no prior cardiac history.

In any event, after initial training and certification, it is important that novice divers progressively gain incremental experience in a relatively controlled manner to improve their knowledge of, and skills in, the diving environment. Careful planning, including assessment of the dive site and conditions, their own and their buddy’s suitability and preparedness for the dive, their equipment, and the conduct of the dive will reduce the likelihood of a serious problem. Further training will enhance knowledge and skills and is encouraged.

As an aside, another important key to safer diving is appropriate supervision, especially in a commercial setting. In Queensland, where there is a large diving industry, the existence of a regulated code of practice (COP) for the conduct of diving in that State appears to have improved safety [16].

### 4.4. Equipment

Improved cylinder pressure gauges became available and increasingly ubiquitous, and, together with increased training, likely contributed to a reduction in out-of-air incidents and subsequent PBT/AGE and/or drowning. However, too many such avoidable incidents still occur and close monitoring of breathing gas, as well as appropriate equipment maintenance, are essential to minimise these.

The introduction of BCDs from the early 1970s has likely helped to reduce primary drowning events. However, divers need to be fully familiar with their BCDs, ensure they are well maintained, and master buoyancy control skills.

Overall, diving equipment has improved considerably over time and equipment-related accidents generally occur because of poor maintenance or unfamiliarity.

### 4.5. Weights and BCDs

There was only a modest increase in divers who had ditched their weights (13 to 17%) and those who were found with inflated or partly inflated BCDs (23 to 34%). These numbers remain far too low and suggest that this potentially life-saving action is still not sufficiently embedded. Adequate training and subsequent periodic reinforcement are necessary to make such actions automatic in the event of a diver in danger of becoming unconscious underwater. This should ensure they ascend to the surface, increasing the chances of survival.

### 4.6. Buddy System

Most divers set off on a dive with a buddy or buddies, although many separate unintentionally, or intentionally, during the dive. It is encouraging to note that the proportion of casualties who were still with a buddy increased between periods, and, despite a poor outcome in these fatalities, the presence of a buddy in a variety of situations has the potential to, and does, mitigate some serious incidents.

### 4.7. Predisposing Factors

The greatest change in PFs between the two periods was the increase in health-related factors. This is predominantly the result of the higher age of the divers and, to some extent, lifestyle factors such as diet and exercise. As mentioned earlier, chronic health conditions are more prevalent in older persons, in particular cardiac disease [13]. The diving environment is arrhythmogenic—more so than many people realise. Cardiac arrhythmias can be triggered by immersion or submersion per se in which buoyancy counters the effect of gravity, re-distributes venous blood from the limbs to the thorax, and results in a substantial increase in the cardiac workload [17,18,19]. Workload is increased further by wearing heavy equipment and restrictive exposure suits, exertion during a dive, breathing resistance from regulators, anxiety, cold-induced vasoconstriction, and the higher oxygen partial pressure at depth. Such factors can combine and increase the likelihood of a cardiac event in a predisposed diver.

In Australia from 1980 to 1999, the proportion of men aged 25–64 years who were obese rose from 9% to 14%, and women from 8% to 20% [20]. Current Australian data reveal a general adult obesity rate of 31%, which rises to 37.4% for those aged 45–54 years [13]. While high, this is substantially lower that the 43% obesity rate in the later scuba victim cohort and suggests that obesity is likely a risk factor for a scuba fatality. The effects of what is often a restrictive wetsuit, excessive weighting to overcome positive buoyancy, and the associated buoyancy changes with changes in depth, as well as the impairment of respiratory function and the increased cardiac demands to overcome these, can present a serious hazard. The relationship between obesity and chronic disease such as hypertension and diabetes, together with cardiac arrhythmias and sudden cardiac death [21,22,23,24,25], should prompt a serious consideration with fitness-to-dive assessments, and possibly a trigger to seek an assessment. Such assessments are currently recommended for some [26] or all [27] divers or prospective divers aged 45 years or older with close emphasis on cardiac health, as well as for younger divers with other potentially contraindicated medical conditions.

### 4.8. Limitations

As with any uncontrolled case series, the collection and analysis of the fatality data are subject to inevitable limitations and uncertainties associated with the investigations. Given that many incidents were unwitnessed, some of the assertions, such as the disabling conditions and the effect of predisposing conditions, can be somewhat speculative.

## 5. Conclusions

**Figure 6:**
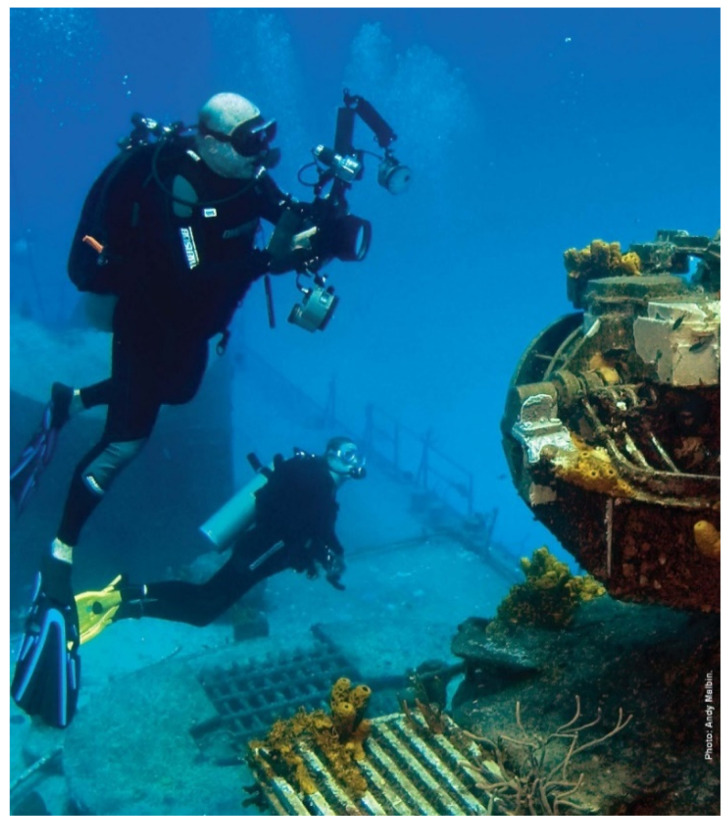
An (older) diver in 2015.

Although there has been no significant increase in average annual deaths between the periods 1972–1999 and 2000–2021, there has been a substantial increase in the casualties’ ages and the proportion of females. The increase in training availably and ubiquitous use of BCDs and cylinder pressure gauges over time has likely been instrumental in reducing primary drowning events. However, the increase in age of participants combined with dietary and exercise factors is accompanied by a higher prevalence of cardiac disease and obesity, leading to a rise in the frequency of cardiac-related fatalities. Improved health education and surveillance, especially of older divers, are essential to reduce such deaths. In addition, improved dive planning, including choice of dive sites and assessment of prevailing and potential conditions, as well as tighter buddy systems, incremental experience, and embedded emergency surfacing protocols, should reduce fatalities, especially in less experienced divers (Figure 6).

## Figures and Tables

**Table 1:** Comparisons of various characteristics of diving fatalities between the periods 1972–1999 and 2000–2021. PBT/AGE = pulmonary barotrauma/arterial gas embolism. BCD = buoyancy control device.

	Period	
Feature	1972–1999	2000–2021
Number of deaths	236	194
Average deaths/year	8.4	8.8
Males (%)	83	76
Median age (yrs)	33	47
Age Range (yrs)	13–70	17–72
Aged ≥45 years (%)	24	57
Uncertified (%)	36	19
In training (%)	8	6
Out of breathing gas (%)	30	25
Solo diving (%)	11	12
With buddy at time (%)	17	27
Weights ditched	13	17
BCD inflated	23	34
*Predisposing factors (%)*		
Health	18	40
Inexperience	40	30
Planning	33	29
Equipment	30	21
*Disabling conditions (%)*		
Asphyxia	47	36
Cardiac	12	26
PBT/CAGE	19	12
Other/Unknown	21	26

## Data Availability

The datasets presented in this article are not readily available because they are largely taken from restricted coronial documents which can only be accessed with particular ethics approvals. Requests to access such data should be directed to the National Coronial Information System (ncis.org.au).
